# Correlation of tumor maximum standardized uptake value from ^18^F-BPA PET and boron neutron capture therapy tumor dose with treatment outcomes in head and neck cancer: a retrospective analysis

**DOI:** 10.1007/s11604-026-02020-7

**Published:** 2026-06-02

**Authors:** Yuki Yoshino, Satoshi Takeno, Teruhito Aihara, Kayako Isohashi, Naonori Hu, Yasukazu Kanai, Akinori Sasaki, Mai Nojiri, Tetsuya Terada, Shinichi Haginomori, Keiji Nihei, Koji Ono

**Affiliations:** 1 Kansai BNCT Medical Center, Educational Foundation of Osaka Medical and Pharmaceutical University, 2-7 Daigakucho, Takatsuki, Osaka, 569-8686 Japan; 2 Department of Radiation Oncology, Osaka Medical and Pharmaceutical University, Osaka, Japan; 3 Department of Radiology, Kyoto Prefectural University of Medicine, Kyoto, Japan; 4 Department of Otorhinolaryngology – Head and Neck Surgery, Osaka Medical and Pharmaceutical University, Osaka, Japan; 5 Department of Radiology, Graduate School of Medicine, The University of Osaka, Osaka, Japan; 6 Institute for Integrated Radiation and Nuclear Science, Kyoto University, Kyoto, Japan; 7 BNCT Joint Clinical Institute, Osaka Medical and Pharmaceutical University, Osaka, Japan

**Keywords:** BNCT, ^18^F-BPA PET/CT, Head and neck cancer, Treatment response

## Abstract

**Purpose:**

Boron neutron capture therapy (BNCT) dose planning conventionally uses a tumor-to-blood (T/B) ratio derived from the tumor maximum standardized uptake value (SUVmax) of fluorine-18-labeled 4-borono-L-phenylalanine (^1^⁸F-BPA) positron emission tomography (PET) (individual model). It remains unclear whether tumor SUVmax or individual model doses predict outcomes in accelerator-based BNCT for head and neck cancer (HNC). We investigated the correlation of ^1^⁸F-BPA PET SUVmax and individual model doses with treatment response.

**Materials and methods:**

We retrospectively analyzed 30 patients with HNC treated with accelerator-based BNCT (2020–2021). The Kaplan–Meier method was used for survival analysis. Dose parameters (Dmax, Dmin, and D80%) were calculated using the uniform model (T/B = 2.5) and the individual model (T/B = Tumor SUVmax/Blood-pool SUV). Correlations between tumor SUVmax or dose parameters and best treatment response [complete response (CR) vs. non-CR] were evaluated using the Mann–Whitney U test.

**Results:**

The objective response rate was 90%, and the CR rate was 50%. CR status strongly predicted superior 2 years overall survival, locoregional control, and progression-free survival (all *p* < 0.05). However, no significant differences were observed between the CR and non-CR groups for tumor SUVmax, uniform model doses, or individual model doses (all *p* > 0.05).

**Conclusion:**

^1^⁸F-BPA PET SUVmax and the conventional individual model did not demonstrate a statistically significant association with CR in patients with HNC after accelerator-based BNCT. These findings question the utility of tumor SUVmax-based dosimetry, suggesting that current models require refinement.

**Supplementary Information:**

The online version contains supplementary material available at https://doi.org/10.1007/s11604-026-02020-7.

## Introduction

Boron neutron capture therapy (BNCT) is a high linear energy transfer (LET) particle therapy based on a boron neutron capture reaction (^10^B (n, α) ^7^Li) [1]. The range of high LET particles from BNCT is less than 10 µm, which is shorter than the diameter of a single cell. Therefore, BNCT can selectively kill tumor cells while minimizing damage to neighboring normal organ cells [2, 3].

For effective BNCT, boron (^10^B) must be selectively incorporated into the tumor. In clinical practice, 4-borono-L-phenylalanine (BPA) is widely used as a boron compound for BNCT [4]. BPA is known to be selectively taken up by tumors via L-type amino acid transporter 1 (LAT1) [5], which is upregulated in various tumors [6]. Based on the results of the JHN002 Phase II study [7], Japanese public health insurance for BNCT using accelerator and BPA was approved in June 2020. According to this regulation, the target tumor must be unresectable, the disease must be locally advanced or locally recurrent head and neck cancer (HNC), and conventional radiotherapy (RT) should be preferred if it is expected to be effective.

BNCT is also a type of radiotherapy, and dose assessment is essential. In BNCT, the total absorbed dose consists of boron neutron doses from boron neutron capture reactions, gamma radiation mixed with the neutron beam, and absorbed doses generated by neutrons interacting with hydrogen and nitrogen in biological tissues. Among these, boron neutron doses account for the majority of the total dose in BNCT. This boron neutron dose is calculated based on the neutron fluence, blood boron concentration, and the tumor/blood (T/B) ratio. Neutron fluence can be calculated using dose calculation software [8]. Blood boron concentration can also be measured through blood sampling. However, currently, it is impossible to determine the T/B ratio of boron concentration during BNCT for individual cases.

Currently, positron emission tomography (PET) using fluorine-18-labeled BPA (^18^F-BPA) is the only clinically available method for speculating BPA distribution in vivo during BNCT. In animal studies, ^18^F-BPA and BPA are reported to have similar pharmacokinetics when administered at the same dosage or under the same protocol [9, 10]. Furthermore, animal studies have reported that the tumor maximum standardized uptake value (SUVmax) of ^18^F-BPA correlates with actual BPA uptake in tumors [11]. This correlation is stronger than that between mean SUV and BPA uptake.

In the field of BNCT, the SUVmax of ^18^F-BPA is used to indicate BPA uptake in tumors [12]. It is common practice to determine the T/B ratio based on tumor SUVmax in “the individual model” [13–18]. When it is difficult to perform ^18^F-BPA PET prior to BNCT, it is a general practice to assume a fixed T/B value for all cases in “the uniform model” based on past research [7, 19–23].

High BPA uptake into tumors is expected to enhance therapeutic efficacy by increasing boron neutron doses. However, few comprehensive statistical analyses have been conducted to verify whether tumor SUVmax derived from ^1^⁸F-BPA accurately reflects the therapeutic efficacy of clinical BNCT in patients with HNC [23]. A previous report has examined tumor doses in the individual model and their relationship to BNCT treatment response [18]. However, that study only analyzed 10 cases and did not use an accelerator. Therefore, to our knowledge, this is the first comprehensive study to verify whether tumor SUVmax from ^18^F-BPA PET and the dose in the individual model could serve as an indicator of the treatment response of clinical BNCT using an accelerator for HNC.

## Materials and methods

### Study design and patients

Among the patients who participated in the designated clinical research project (title: “Exploration of Threshold SUV Values for Differentiating Malignant Tumors Using ^18^F-BPA PET/Computed Tomography (CT)” (JRCSTs051190116)), 30 patients diagnosed with HNC through histopathological examination and who received BNCT at our institution as Japanese insurance-covered treatment between June 2020 and October 2021 were included in this analysis. We limited eligible patients to those who were expected to receive curative dose treatment by a pre-treatment plan using diagnostic CT. In this study, based on our previous study [22, 24], the curative therapy was defined as the dose of the 80% tumor volume (D_80%_) more than 20 Gray X-ray equivalent dose (Gy-Eq) using the uniform model (T/B ratio = 2.5).

The primary endpoint was to assess the correlation of treatment response after BNCT with either tumor SUVmax on ^1^⁸F-BPA PET or the tumor dose. The tumor dose was calculated using the T/B ratio, which was defined as tumor SUVmax/blood-pool SUV for the individual model and 2.5 for the uniform model.

### ^18^F-BPA PET/CT protocol and analysis

^18^F-BPA was synthesized due to the direct radiofluorination of BPA [25] using the MPS-200 synthesizer (Sumitomo Heavy Industries Ltd., Tokyo, Japan) in our institution. Patients were injected with a standard dose of 4 MBq/kg of good manufacturing practice-grade purified ^18^F-BPA, ranging from 185 to 370 MBq according to the institutional guidelines for the clinical use of radiopharmaceuticals. One hour later, a whole-body scan was performed, typically requiring 16 min for eight bed positions, followed by an additional local scan lasting 10 min. SUV was calculated as radioactivity measured by PET (Bq/g) divided by injected radioactivity per body weight (Bq/g), where the specific gravity of the tissue was assumed as 1.0 (g/mL).

### BNCT planning and treatment

The gross tumor volume (GTV) and organs at risk and the generation of the treatment plans for BNCT were contoured using RayStation® version 9A (Raysearch Laboratories AB, Stockholm, Sweden). The dose was calculated using the NeuCure Dose Engine® (Sumitomo Heavy Industries Ltd.) [8]. The treatment plans were calculated using a compound biological effectiveness (CBE) factor of 3.8, 4.9, 2.5, and 1.35 at the tumor, mucosa, skin, and other normal tissues, respectively. The CBE factor is similar to the relative biological effectiveness factor in BNCT [26–29]. The prescribed dose was set to 15, 12, 9, and 5 Gy-Eq for the skin, pharyngeal mucosa, brain, and eyes, respectively.

Borofalan (^10^B), a BPA-based drug, was the only drug approved by the Japanese regulatory agency. Borofalan was administered according to Ono’s method [30]: 200 mg/kg/h for the first 2 h before irradiation, followed by 100 mg/kg/h once the irradiation began, to maintain the boron concentration during irradiation. The blood ^10^B concentration was measured via inductively coupled plasma optical emission spectrometry (ICP-OES) on an Agilent 5110 ICP-OES (Agilent Technologies, Santa Clara, CA, USA). The post-treatment plan was created based on the average of the blood ^1^⁰B concentrations measured before and after neutron irradiation. We analyzed post-treatment plan data in this study.

### Assessment

TNM classification and clinical stage before BNCT were determined based on the Union for International Cancer Control (UICC) TNM classification 8th Edition.

Post-treatment follow-up was performed at our institution at 7 days and every 1–3 months after BNCT, whenever possible. Treatment response was clinically evaluated based on the Response Evaluation Criteria in Solid Tumors version 1.1. Among the efficacy data collected over the 2 years following BNCT, the best response was adopted as the treatment response.

### Statistical analyses

The Kaplan–Meier method was used to estimate median overall survival (OS), locoregional control (LRC), and progression-free survival (PFS). The LRC duration was defined as the period from the date of BNCT irradiation to the date of disease progression at the irradiated site, whereas the PFS duration was defined as the period from the date of BNCT irradiation to the date of disease progression or death. The log-rank test was used to compare the two groups (CR vs. non-CR).

About treatment response (CR vs. non-CR), patients’ characteristics such as age, sex, histology, past RT, past surgery, prior systemic therapy, T stage, N stage, systemic therapy after BNCT before progression, and GTV volume were analyzed using two-sided univariate logistic regression. For continuous variables, the cut-off value was selected as the median.

We examined each Spearman’s rho for tumor SUVmax, the maximum dose of tumors (Dmax), the minimum dose of tumors (Dmin), and D80% from the individual model and defined strong correlation as Spearman’s ρ > 0.6. Tumor SUVmax and Dmin, Dmax, and D80% from the uniform model or the individual model were compared between the CR group and the non-CR group using a two-sided Mann–Whitney U test.

Statistical significance was set at a *p* value < 0.05. All statistical analyses were performed using GraphPad PRISM 10, version 10.4.1 (GraphPad Software, Boston, MA, USA).

## Results

Table 1 shows the patient characteristics. The median age was 73, with a slight male predominance. The primary diseases were varied. The common histology was squamous cell carcinoma (SqCC; 83%). Prior to BNCT, RT, surgery, and systemic therapy were administered in 70%, 60%, and 57% of cases, respectively. Only one patient had an M-stage, but the cancer lesions were limited to sternal metastases, making it a case where curative treatment with BNCT was expected. Three patients underwent systemic therapy after BNCT before progression. The median GTV volume was 10.3 cc, and the median tumor SUVmax of ^18^F-BPA was 4.2. The median values for Dmax, Dmin, and D80% for the GTV in the uniform model were 36, 20, and 27 Gy-Eq, respectively (Table 2). The median values for Dmax, Dmin, and D80% relative to the GTV in the individual model were 44, 27, and 33 Gy-Eq, respectively. In the uniform model, there were four cases with D80 < 20 Gy-Eq. In the individual model, there was one case with D80 < 20 Gy-Eq.

**Table 1:** Patient characteristics

Characteristics	All (n = 30)	
	n	%
Median age, years (range)	73 (39–90)	
Sex, number		
Male	17	57
Female	13	43
Primary site, number		
Oral (tongue, gingiva, buccal mucosa)	8	27
Pharynx (naso, oro, hypo)	7	23
External auditory canal	4	13
Parotid gland	3	10
Others (larynx, unknown, cervical esophagus, sinus, skin)	8	27
Histology, number		
SqCC	25	83
Others	5	17
Past RT, number	21	70
Past surgery, number	18	60
Prior systemic therapy	17	57
T stage, number		
0	13	43
1	1	3
2	1	3
3	4	13
4	11	37
N stage, number		
0	16	53
1	4	13
2	2	7
3	8	27
M stage, number		
0	29	97
1	1	3
Systemic therapy after BNCT before progression, number	3	10
Median GTV volume, cc (range)	10.3 (1.15–235)	
Median days between ^18^F-BPA and BNCT	13 (1–62)	
Tumor SUVmax from ^18^F-BPA (range)	4.2 (2.3–13)	

SqCC, squamous carcinoma; RT, radiotherapy; BNCT, boron neutron capture therapy; GTV, gross tumor volume; ^18^F-BPA, fluorine-18-labeled 4-borono-L-phenylalanine; SUVmax, maximum standardized uptake value

**Table 2:** Dose of GTV for patients with head and neck cancer

GTV dose, Gy-Eq	Uniform model	Individual model
Median maximum dose, (range)	36 (22–54)	44 (23–157)
Median minimum dose, (range)	20 (8.1–28)	27 (11–93)
Median dose of 80% GTV volume, (range)	27 (17–42)	33 (18–132)

GTV, gross tumor volume; Gy-Eq, Gray X-ray equivalent dose

**Fig. 1:**
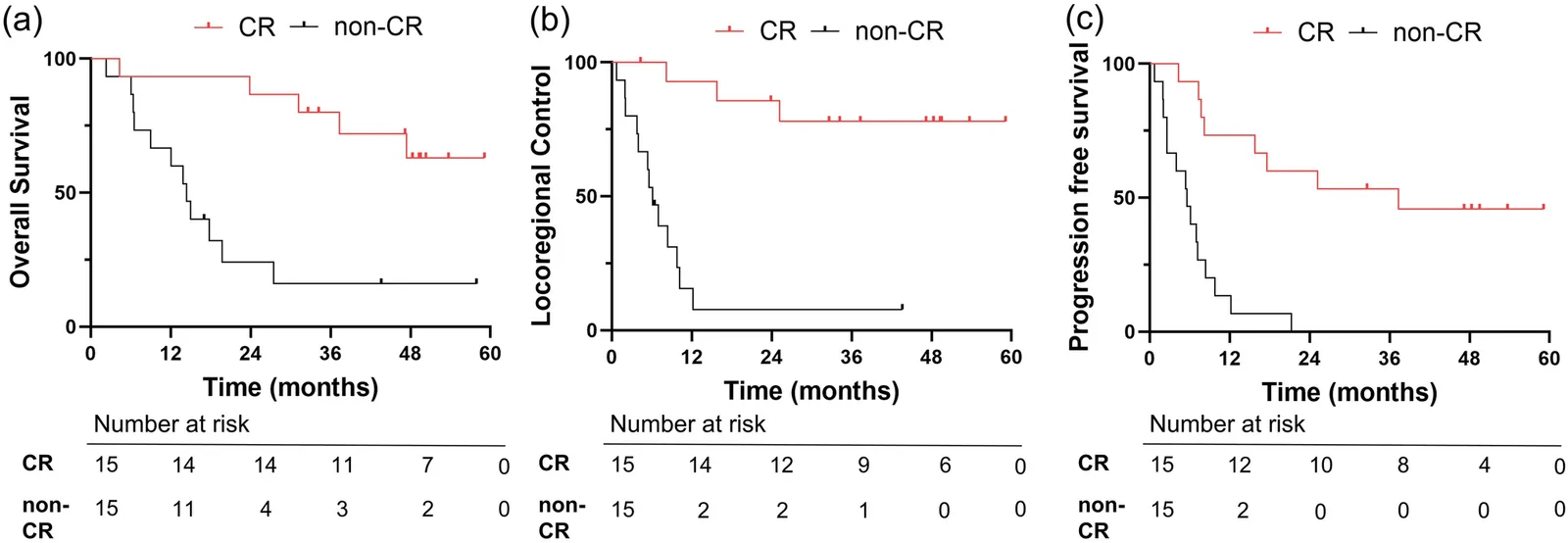
Survival outcomes comparing CR and non-CR cases. **a** Overall survival. **b** Locoregional control. **c** Progression-free survival. CR = complete response

The objective response [partial response (PR) + CR] rate among the 30 patients analyzed was 90% (27/30), and the CR rate was 50% (15/30). The objective response rate is an imbalanced dataset with a small number of non-objective overall response cases, which makes comparative analysis difficult. Therefore, we only investigated the correlation of treatment efficacy with respect to the CR rate. In patients who achieved CR after BNCT, the 2 years OS, LRC, and PFS rates were 93%, 80%, and 67%, respectively, which were significantly higher than those in patients with non-CR (27%, 13%, and 0%, respectively; all *p* < 0.05; Fig. 1). No significant differences in patient background characteristics were observed regarding treatment response (CR vs. non-CR) using two-sided univariate logistic regression (Table 3).

**Table 3:** Univariate logistic regression of patient characteristics

	Patients	Treatment response		*p* value	Odds ratio	95% CI
	(n)	CR	non-CR			
Age				> 0.9999	1	0.2345–4.2640
< 70	14	7	7			
> 70	16	8	8			
Sex				0.2726	0.4375	0.09422–1.87500
Male	17	7	10			
Female	13	8	5			
Histology				0.6262	1.625	0.2305–14.0000
SqCC	25	13	12			
Others	5	2	3			
Past RT				0.4129	2	0.3908–11.8000
Yes	21	13	8			
No	9	2	7			
Past surgery				0.4577	0.5714	0.1243–2.4780
Yes	18	8	10			
No	12	7	5			
Prior systemic therapy				0.7128	0.7619	0.1739–3.2540
Yes	17	8	9			
No	13	7	6			
T stage				0.7128	1.313	0.3073–5.7500
0	13	7	6			
non 0	17	8	9			
N stage				0.7128	1.313	0.3073–5.7500
0	17	9	8			
non 0	13	6	7			
Systemic therapy after BNCT before progression				0.5501	0.1847–49.6300	-1.689–3.905
Yes	3	2	1			
No	27	13	14			
GTV volume, (cc)				0.2766	0.4444	0.09785–1.87800
< 10	15	9	6			
> 10	15	6	9			

SqCC, squamous carcinoma; RT, radiotherapy; BNCT, boron neutron capture therapy; GTV, gross tumor volume; CI, confidence interval

**Fig. 2:**
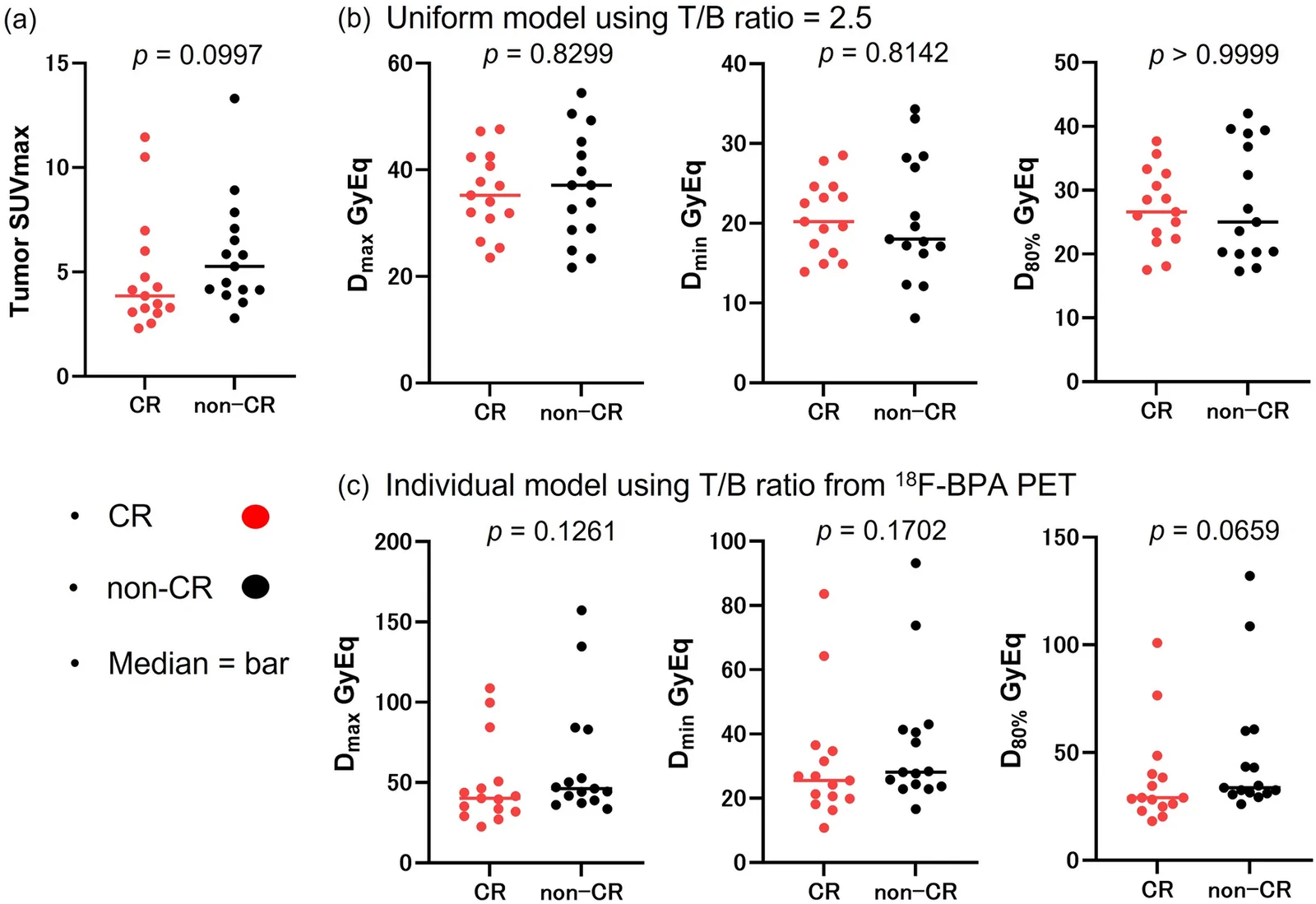
Comparison between the CR and non-CR groups using a two-sided Mann–Whitney U test. **a** Comparison of tumor SUVmax. **b** Comparison of Dmin, Dmax, and D80% from the uniform model. **c** Comparison of Dmin, Dmax, and D80% from the uniform model and the individual model. CR = complete response; Dmax = the maximum dose of the tumor; Dmin = the minimum dose of the tumor; D_80%_ = dose received by 80% of the tumor volume; SUVmax = maximum standardized uptake value

The Dmax, Dmin, and D80% values for each model showed a significantly strong correlation with tumor SUVmax (all Spearman’s ρ > 0.6, all *p* < 0.05). No significant difference was observed between the CR group and the non-CR group regarding tumor SUVmax (Fig. 2a). No significant differences were observed between the CR group and the non-CR group for Dmax, Dmin, and D80% in both the uniform model and the individual model (Fig. 2b and c). A summary of these results is presented in Table 4. Furthermore, we compared SUVmax, Dmax, Dmin, and D80% in the individual model between the CR and non-CR groups, stratified by the median time interval of 13 days between ^1^⁸F-BPA PET and BNCT (≤ 13 days vs. > 13 days). No statistically significant differences were observed in any of these stratified analyses (Online Resource 1).

**Table 4:** Summary of comparisons between the CR and non-CR groups using a two-sided Mann–Whitney U test

Parameter	Median value		Hodges–Lehmann estimate	*p* value
Uniform model	CR	non-CR		
D_max_, Gy-Eq	35.2	37.1	0.7	0.8299
D_min_, Gy-Eq	20.2	18	− 0.6	0.8142
D_80%_, Gy-Eq	26.6	25	0	> 0.9999
Individual model	CR	non-CR		
SUV_max_	3.85	5.27	1.07	0.0997
D_max_, Gy-Eq	40.3	46.3	7.1	0.1261
D_min_, Gy-Eq	25.5	28.1	4.8	0.1702
D_80%_, Gy-Eq	29.1	33.7	6.1	0.0659

CR, complete response; Gy-Eq, Gray X-ray equivalent dose; SUVmax, maximum standardized uptake value; non-CR, non–complete response

## Discussion

Although treatment response was simplified by dichotomizing patients into CR and non-CR groups (primarily to avoid imbalances in response categories), we consider this approach clinically relevant because the distinction between these groups was stark and clearly reflected differences in prognosis in this study and the previous study [23].

This study was conducted based on the concept that BPA uptake and dose are the most significant factors influencing the therapeutic efficacy of BNCT. Therefore, patient background factors such as primary tumor site, stage, and tumor size were not standardized. However, no significant differences in treatment response were observed based on patient background. The differences in patient background are considered to have had little impact on the observed differences in treatment response with ^18^F-BPA.

The T/B ratio derived from ^18^F-BPA-PET is calculated from tumor SUVmax. This is consistent with the correlation between individual model dose and tumor SUVmax. Although higher tumor SUVmax values are expected to correlate with higher boron concentrations, tumor SUVmax was not an effective indicator of BNCT treatment efficacy. In BNCT, a favorable therapeutic effect cannot be achieved without a sufficient neutron dose. Therefore, it is difficult to predict treatment efficacy based solely on tumor SUVmax. A previous study has also reported in supplementary materials that tumor SUVmax did not correlate with treatment efficacy [23]. On the other hand, the tumor dose calculated using the uniform model also failed to reflect treatment efficacy. This is thought to be because individual differences in BPA uptake in tumors are not accounted for. In fact, the other previous studies have not shown that higher tumor doses calculated using the uniform model lead to significantly better treatment efficacy in curative settings, either [7, 22].

We examined whether a correlation could be observed between the dose and therapeutic effect in the individual model, considering both aspects of neutron distribution and BPA uptake. Contrary to our expectations, the individual model did not serve as an indicator reflecting the CR rate. This could be due to various reasons. First, as reported in many previous studies [13–18], this conventional individual model calculates the T/B ratio from tumor SUVmax. However, this method assumes BPA is distributed uniformly within the tumor. In actual tumors, ^18^F-BPA is often distributed heterogeneously, and some tumors include necrotic areas (Online Resource 2). Furthermore, relying on a single-point measurement such as SUVmax is inherently susceptible to variability in region of interest placement and measurement error, which may further limit its reliability as a surrogate for overall tumor boron distribution. A previous report indicates that in individual models, CR rates are significantly higher (40%) at D80 > 20 Gy-Eq [24]. In our study, all cases met the curative therapy criteria during the pre-treatment plan, and in the post-treatment plan dose assessment, most cases met the criteria using both the uniform and individual models. After that requirement was met, the focus shifted to whether therapeutic efficacy increases as the dose rises. However, the tumor doses in this conventional individual model did not reflect treatment response. Furthermore, previous studies have reported that tumor doses calculated using this conventional individual model did not reflect treatment efficacy, although this analysis used non-accelerator and involved fewer cases (n = 10) than our study [18]. In other words, the tumor dose calculated using this conventional individual model may not be precise enough to determine the actual therapeutic effect of BNCT. This raises questions about the conventional approach in BNCT for HNC, where BPA uptake has been evaluated using tumor SUVmax from ^18^F-BPA and tumor dose calculated based on that value. To evaluate tumor dose, it may be necessary to construct a more accurate individual model that reflects the heterogeneous distribution of ^18^F-BPA.

Second, this study needs to consider the possibility that ^18^F-BPA does not accurately reflect BPA uptake or distribution during BNCT. In clinical practice, ^18^F-BPA is administered as a single injection of a small amount, whereas BPA is administered as a continuous injection in a large amount over approximately 2 h during treatment. However, no study exists—neither in humans nor in animal experiments—that demonstrates identical biodistribution between ^18^F-BPA and BPA when all factors—administration timing, dosage, and protocol—differ. Watanabe et al. reported that, in an animal model of SqCC (the most common type of HNC) [9], there was no difference in BPA uptake when dosages were equal, but protocols differed. However, this result was obtained using subcutaneous injection, not intravenous injection. Although the administration timing and protocol (single-shot intravenous injection) were the same, Hanaoka et al. demonstrated a correlation between the biodistribution of ^18^F-BPA and BPA, even with different doses [10]. Furthermore, Watabe et al. showed a correlation between tumor SUVmax from ^18^F-BPA and BPA uptake despite slightly different administration protocols (^18^F-BPA single shot vs. BPA administered over 2 min) [11]. However, these are experimental results obtained using glioma-bearing animals. It may not be possible to conclude that the same holds true for SqCC models. Therefore, it may also be necessary to explore methods for visualizing the biodistribution of BPA during BNCT other than ^18^F-BPA PET.

This analysis revealed no significant differences in treatment response based on ^18^F-BPA PET data. However, the limitations of this study are the retrospective nature of the analysis and the small sample size, which may have precluded a definitive analysis. Additionally, dichotomizing responses into CR and non-CR due to the small number of SD/PD cases (n = 3) might have masked subtle dose–response gradients within the non-CR group. Therefore, we believe that, moving forward, accumulating more cases for the prospective analysis is necessary. Furthermore, future analyses within groups with more similar backgrounds may be desirable, even though patient background did not significantly influence treatment response in this study. Additionally, measures to minimize the time gap between ^18^F-BPA PET and BNCT administration must be devised.

In conclusion, this study is the first comprehensive report to verify whether tumor SUVmax from ^18^F-BPA PET and the dose in the conventional individual model could serve as an indicator of the treatment response of clinical BNCT using an accelerator for HNC. Contrary to our expectations, however, the tumor doses derived from tumor SUVmax of ^18^F-BPA PET and the conventional individual model based on it did not significantly reflect the CR rate. This raises questions about the practice in the BNCT field of calculating tumor dose for BNCT based on tumor SUVmax from ^18^F-BPA PET. At the very least, the individual model still requires significant refinement, and further research is needed.

## Supplementary Information

Below is the link to the electronic supplementary material.

Supplementary file1 (DOCX 658 KB)

## References

[1] Locher GL. Biological effects and therapeutic possibilities of neutron. AJR Am J Roentgenol. 1936;36:1–3.

[2] Coderre JA, Morris GM. The radiation biology of boron neutron capture therapy. Radiat Res. 1999;151:1–18. 9973079

[3] International Atomic Energy Agency. Advances in boron neutron capture therapy. Vienna: International Atomic Energy Agency; 2023.

[4] Mishima Y, Honda C, Ichihashi M, Obara H, Hiratsuka J, Fukuda H, et al. Treatment of malignant melanoma by single thermal neutron capture therapy with melanoma-seeking 10B-compound. Lancet. 1989;2:388–9. 10.1016/s0140-6736(89)90567-9 2569577

[5] Wongthai P, Hagiwara K, Miyoshi Y, Wiriyasermkul P, Wei L, Ohgaki R, et al. Boronophenylalanine, a boron delivery agent for boron neutron capture therapy, is transported by ATB0,+, LAT1 and LAT2. Cancer Sci. 2015;106:279–86. 10.1111/cas.12602 PMC4376436 25580517

[6] Hayashi K, Anzai N. Novel therapeutic approaches targeting L-type amino acid transporters for cancer treatment. World J Gastrointest Oncol. 2017;9:21–9. 10.4251/wjgo.v9.i1.21 PMC5241523 28144396

[7] Hirose K, Konno A, Hiratsuka J, Yoshimoto S, Kato T, Ono K, et al. Boron neutron capture therapy using cyclotron-based epithermal neutron source and borofalan (10B) for recurrent or locally advanced head and neck cancer (JHN002): an open-label phase II trial. Radiother Oncol. 2021;155:182–7. 10.1016/j.radonc.2020.11.001 33186684

[8] Hu N, Tanaka H, Kakino R, Yoshikawa S, Miyao M, Akita K, et al. Evaluation of a treatment planning system developed for clinical boron neutron capture therapy and validation against an independent Monte Carlo dose calculation system. Radiat Oncol. 2021;16:243. 10.1186/s13014-021-01968-2 PMC8709965 34952608

[9] Watanabe T, Hattori Y, Ohta Y, Ishimura M, Nakagawa Y, Sanada Y, et al. Comparison of the pharmacokinetics between L-BPA and L-FBPA using the same administration dose and protocol: a validation study for the theranostic approach using [18F]-L-FBPA positron emission tomography in boron neutron capture therapy. BMC Cancer. 2016;16:859. 10.1186/s12885-016-2913-x PMC5100278 27821116

[10] Hanaoka K, Watabe T, Naka S, Kanai Y, Ikeda H, Horitsugi G, et al. FBPA PET in boron neutron capture therapy for cancer: prediction of (10)B concentration in the tumor and normal tissue in a rat xenograft model. EJNMMI Res. 2014;4:70. 10.1186/s13550-014-0070-2 PMC4293470 25621196

[11] Watabe T, Hanaoka K, Naka S, Kanai Y, Ikeda H, Aoki M, et al. Practical calculation method to estimate the absolute boron concentration in tissues using 18F-FBPA PET. Ann Nucl Med. 2017;31:481–5. 10.1007/s12149-017-1172-5 PMC5486508 28439784

[12] Imahori Y, Ueda S, Ohmori Y, Sakae K, Kusuki T, Kobayashi T, et al. Positron emission tomography-based boron neutron capture therapy using boronophenylalanine for high-grade gliomas: part II. Clin Cancer Res. 1998;4:1833–41. 9717809

[13] Kato I, Ono K, Sakurai Y, Ohmae M, Maruhashi A, Imahori Y, et al. Effectiveness of BNCT for recurrent head and neck malignancies. Appl Radiat Isot. 2004;61:1069–73. 10.1016/j.apradiso.2004.05.059 15308194

[14] Aihara T, Hiratsuka J, Morita N, Uno M, Sakurai Y, Maruhashi A, et al. First clinical case of boron neutron capture therapy for head and neck malignancies using 18F-BPA PET. Head Neck. 2006;28:850–5. 10.1002/hed.20418 16721735

[15] Suzuki M, Sakurai Y, Nagata K, Kinashi Y, Masunaga S, Ono K, et al. Impact of intra-arterial administration of boron compounds on dose-volume histograms in boron neutron capture therapy for recurrent head-and-neck tumors. Int J Radiat Oncol Biol Phys. 2006;66:1523–7. 10.1016/j.ijrobp.2006.07.1373 17056200

[16] Ariyoshi Y, Miyatake SI, Kimura Y, Shimahara T, Kawabata S, Nagata K, et al. Boron neutron capture therapy using epithermal neutrons for recurrent cancer in the oral cavity and cervical lymph node metastasis. Oncol Rep. 2007;18:861–6. 17786347

[17] Suzuki M, Kato I, Aihara T, Hiratsuka J, Yoshimura K, Niimi M, et al. Boron neutron capture therapy outcomes for advanced or recurrent head and neck cancer. J Radiat Res. 2014;55:146–53. 10.1093/jrr/rrt098 PMC3885131 23955053

[18] Aihara T, Hiratsuka J, Fukumitsu N, Ishikawa H, Morita N, Kumada H, et al. Evaluation of fluorine-18-labeled boronophenylalanine-positron emission tomography imaging for the assessment of boron neutron capture therapy in patients with recurrent head and neck squamous cell carcinoma. Otolaryngol. 2016;6:277.

[19] Kankaanranta L, Seppälä T, Koivunoro H, Saarilahti K, Atula T, Collan J, et al. Boron neutron capture therapy in the treatment of locally recurred head and neck cancer. Int J Radiat Oncol Biol Phys. 2007;69:475–82. 10.1016/j.ijrobp.2007.03.039 17689034

[20] Kankaanranta L, Seppälä T, Koivunoro H, Saarilahti K, Atula T, Collan J, et al. Boron neutron capture therapy in the treatment of locally recurred head-and-neck cancer: final analysis of a phase I/II trial. Int J Radiat Oncol Biol Phys. 2012;82:e67-75. 10.1016/j.ijrobp.2010.09.057 21300462

[21] Haapaniemi A, Kankaanranta L, Saat R, Koivunoro H, Saarilahti K, Mäkitie A, et al. Boron neutron capture therapy in the treatment of recurrent laryngeal cancer. Int J Radiat Oncol Biol Phys. 2016;95:404–10. 10.1016/j.ijrobp.2015.11.010 26797537

[22] Takeno S, Yoshino Y, Aihara T, Higashino M, Kanai Y, Hu N, et al. Preliminary outcomes of boron neutron capture therapy for head and neck cancers as a treatment covered by public health insurance system in Japan: real-world experiences over a 2-year period. Cancer Med. 2024;13:e7250. 10.1002/cam4.7250 PMC11145025 38826090

[23] Hirose K, Sato M. Clinical results and prognostic factors in boron neutron capture therapy for recurrent squamous cell carcinoma of the head and neck under the Japan National Health Insurance System: a retrospective study of the initial 47 patients. Int J Radiat Oncol Biol Phys. 2024;120:796–804. 10.1016/j.ijrobp.2024.03.041 38580084

[24] Aihara T, Hiratsuka J, Higashino M, Kawata R, Ono K. BNCT for head and neck cancer; current and future (in Japanese). Bio Clinica. 2019;34:433–40.

[25] Ishiwata K, Ido T, Mejia AA, Ichihashi M, Mishima Y. Synthesis and radiation dosimetry of 4-borono-2-[18F]fluoro-d,l-phenylalanine: a target compound for PET and boron neutron capture therapy. Int J Radiat Appl Instrum A. 1991;42:325–8. 10.1016/0883-2889(91)90133-l 1850716

[26] Hiratsuka J, Fukuda H, Kobayashi T, Karashima H, Yoshino K, Imajo Y, et al. The relative biological effectiveness of 10B-neutron capture therapy for early skin reaction in the hamster. Radiat Res. 1991;128:186–91. 1947014

[27] Coderre JA, Makar MS, Micca PL, Nawrocky MM, Liu HB, Joel DD, et al. Derivations of relative biological effectiveness for the high-let radiations produced during boron neutron capture irradiations of the 9l rat gliosarcoma in vitro and in vivo. Int J Radiat Oncol Biol Phys. 1993;27:1121–9. 10.1016/0360-3016(93)90533-2 8262837

[28] Fukuda H, Hiratsuka J, Honda C, Kobayashi T, Yoshino K, Karashima H, et al. Boron neutron capture therapy of malignant melanoma using 10B-paraboronophenylalanine with special reference to evaluation of radiation dose and damage to the normal skin. Radiat Res. 1994;138:435–42. 8184019

[29] Coderre JA, Morris GM, Kalef-Ezra J, Micca PL, Ma R, Youngs K, et al. The effects of boron neutron capture irradiation on oral mucosa: evaluation using a rat tongue model. Radiat Res. 1999;152:113–8. 10409319

[30] Ono K. Two-step rate infusion of 4-borono-L-phenylalanine (BPA) solution in BPA-BNCT (Ono’s method). In: *Advances in boron neutron capture therapy*. Vienna: International Atomic Energy Agency; 2023.

