# Supplementary material for: Correlation of tumor maximum standardized uptake value from 18F-BPA PET and boron neutron capture therapy tumor dose with treatment outcomes in head and neck cancer: a retrospective analysis
Source: Jpn J Radiol. 2026 Jun 2;44(10):1845–53. doi: 10.1007/s11604-026-02020-7 (PMC13623982; doi:10.1007/s11604-026-02020-7)
Supplement: Supplementary file 1 [file 11604_2026_2020_MOESM1_ESM.docx]

**Journal name:** *Japanese Journal of Radiology*

**Title:** Correlation of Tumor Maximum Standardized Uptake Value from ^18^F-BPA PET and Boron Neutron Capture Therapy Tumor Dose with Treatment Outcomes in Head and Neck Cancer: A Retrospective Analysis

Yuki Yoshino ^a,b,c*^, Satoshi Takeno ^a,b^, Teruhito Aihara ^a,d^, Kayako Isohashi ^e^, Naonori Hu ^a,f^, Yasukazu Kanai ^a,g^, Akinori Sasaki ^a^, Mai Nojiri ^a^, Tetsuya Terada ^d^, Shinichi Haginomori ^d^, Keiji Nihei ^a,b^, Koji Ono ^a,g^

^a^ Kansai BNCT Medical Center, Osaka Medical and Pharmaceutical University, Osaka, Japan

^b^ Department of Radiation Oncology, Osaka Medical and Pharmaceutical University, Osaka, Japan

^c^ Department of Radiology, Kyoto Prefectural University of Medicine, Kyoto, Japan

^d^ Department of Otorhinolaryngology – Head and Neck Surgery, Osaka Medical and Pharmaceutical University, Osaka, Japan

^e^ Department of Radiology, Graduate School of Medicine, The University of Osaka, Osaka, Japan

^f^ Institute for Integrated Radiation and Nuclear Science, Kyoto University, Kyoto, Japan

^g^ BNCT Joint Clinical Institute, Osaka Medical and Pharmaceutical University, Osaka, Japan

***Corresponding Author:**

Email: [yuki.yoshino@ompu.ac.jp](mailto:yuki.yoshino@ompu.ac.jp)

**Online Resource 1.** Comparison between the CR and non-CR groups using a two-sided Mann–Whitney U test (individual model). The interval time refers to the number of days between ^18^F-BPA and BNCT. **(a)** Comparison of tumor SUVmax, Dmin, Dmax, and D80% for duration time ≤ 13 days. **(b)** Comparison of tumor SUVmax, Dmin, Dmax, and D80% for interval time > 13 days.

Abbreviations: CR = complete response; Dmax = the maximum dose of the tumor; Dmin = the minimum dose of the tumor; D_80%_ = dose received by 80% of the tumor volume; SUVmax = maximum standardized uptake value.

**Online Resource 2.** Tumor images of ^18^F-BPA PET/CT before BNCT with GTV volumes. The area outlined in green indicates the tumor. The higher the brightness, the more ^18^F-BPA accumulates. **(a)** All CR cases. **(b)** All non-CR cases. **(c)** Comparison of GTV volumes between the CR group and non-CR group using a two-sided Mann–Whitney U test.

Abbreviations: ^18^F-BPA PET/CT = fluorine-18-labeled 4-borono-L-phenylalanine positron emission tomography/computed tomography; CR = complete response; GTV = gross tumor volume; BNCT = boron neutron capture therapy.
